# An Integrated mRNA and microRNA Expression Signature for Glioblastoma Multiforme Prognosis

**DOI:** 10.1371/journal.pone.0098419

**Published:** 2014-05-28

**Authors:** Jie Xiong, Zhitong Bing, Yanlin Su, Defeng Deng, Xiaoning Peng

**Affiliations:** 1 Department of Statistics, College of Mathematics and Computer Science, Hunan Normal University, Changsha, China; 2 Department of Molecular Epidemiology, College of Medicine, Hunan Normal University, Changsha, China; 3 Department of Computational Physics, Institute of Modern Physics of Chinese Academy of Sciences, Lanzhou, China; 4 Department of Cancer Biology, The University of Texas MD Anderson Cancer Center, Houston, Texas, United States of America; University of California, Los Angeles, United States of America

## Abstract

Although patients with Glioblastoma multiforme (GBM) have grave prognosis, significant variability in patient outcome is observed. The objective of this study is to identify a molecular signature for GBM prognosis. We subjected 355 mRNA and microRNA expression profiles to elastic net-regulated Cox regression for identification of an integrated RNA signature for GBM prognosis. A prognostic index (PI) was generated for patient stratification. Survival comparison was conducted by Kaplan-Meier method and a general multivariate Cox regression procedure was applied to evaluate the independence of the PI. The abilities and efficiencies of signatures to predict GBM patient outcome was assessed and compared by the area under the curve (AUC) of the receiver-operator characteristic (ROC). An integrated RNA prognostic signature consisted by 4 protective mRNAs, 12 risky mRNAs, and 1 risky microRNA was identified. Decreased survival was associated with being in the high-risk group (hazard ratio = 2.864, P<0.0001). The prognostic value of the integrated signature was validated in five independent GBM expression datasets (n = 201, hazard ratio = 2.453, P<0.0001). The PI outperformed the known clinical factors, mRNA-only, and miRNA-only prognostic signatures for GBM prognosis (area under the ROC curve for the integrated RNA, mRNA-only, and miRNA-only signatures were 0.828, 0.742, and 0.757 at 3 years of overall survival, respectively, P<0.0001 by permutation test). We describe the first, to our knowledge, robust transcriptome-based integrated RNA signature that improves the current GBM prognosis based on clinical variables, mRNA-only, and miRNA-only signatures.

## Introduction

Glioblastoma multiforme (GBM) is the most common and lethal type of primary brain tumor in adults. The median overall survival (OS) time of patients with GBM ranges from 12 to 17 months [1]. Demographic and clinical variables such as patient age at diagnosis, preoperative Karnofsky performance score (KPS), and adjuvant therapy are predictive of the OS of patients with GBM [2], [3]. Due to the pathological and clinical heterogeneity of GBMs, standard therapeutic protocols based on maximal safe surgical resection followed by radiation and chemotherapy with temozolomide do not greatly ameliorate the poor survival of GBM patients [1].

In recent years, efforts have been made to better understand GBMs through basic researches (molecular and genetic) [4]. Generally, individual gene or protein assays used alone or in combination with histological features do not predict the survival of GBM patients and are unable to guide therapeutic decisions. Although the genes and proteins themselves may play a role in the biology of GBM, their utility as diagnostic or prognostic markers is not yet clear, perhaps due to molecular heterogeneity within the tumor groupings.

By characterizing genetic alterations, epigenetic alterations, and the expression of cancer genomes, The Cancer Genome Atlas (TCGA) project has provided a comprehensive way to understand GBM [5]. As the number of GBM samples in this project grows, the opportunities to identify prognostic molecular signatures for patients with GBM are increasing. Intrinsic gene expression profiles of GBM may be a better predictor of patient survival than histological features are [6]. Several groups have investigated molecular prognostic signatures and molecular subtypes of GBM based on the expression of GBM genomes [7]–[10]. Kawaguchi et al. [7] described an expression profiling study of a panel of 32 patients with GBM and identified 25 mRNAs that might predict their OS. Arimappamagan et al. [8] profiled the mRNA profiles of 123 GBM patients by reverse-transcriptase polymerase chain reaction and identified 14 mRNAs that could predict survival in GBM patients. The group of Srinivasan et al. [9] was the first to propose a microRNA (miRNA) signature, which consisted of 10 miRNAs and could accurately predict GBM patient survival. These studies mainly focused on mRNA profiles or miRNA profiles of GBM genomes independently, and the prognostic signatures were quite different due to a low sample size or the use of an inappropriate regression method for parameter estimation.

In RNA expression microarray analysis, there is a so-called “curse of dimensionality” problem in that the number of genes is much larger than the number of samples [11]. In this setting, ordinary regression is subject to over-fitting and instable coefficients, and stepwise variable selection methods do not scale well [12], [13]. Regression by penalization methods has been successfully adapted to high-dimensional multiple genomic datasets and outperforms univariate and multivariate regression methods [14]. At present, the most commonly used penalization methods are ridge regression, Least Absolute Shrinkage and Selection Operator (LASSO) regression and a hybrid of these (elastic net regression); all three methods are based on penalizing the L1 norm, the L2 norm, and both the L1 norm and L2 norm with tuning parameters. Although the traditional Cox proportional hazards model is widely used to discover cancer prognostic factors, it is not appropriate for the genomic setting due to the high dimensionality and colinearity. Several groups have proposed to combine the Cox regression model with the elastic net dimension reduction method to select survival-correlated genes within a high-dimensional expression dataset and have made available the associated computation procedures [15]–[17].

In the current study, we subjected the integrated mRNA and miRNA profiles of GBM patients to elastic net-regulated Cox regression analysis and identified an integrated prognostic RNA signature that can predict the OS of GBM patients. The robustness and reproducibility of the prognostic value of the RNA signature was validated in independent external datasets and compared with the prognostic value of previously reported mRNA-only and miRNA-only signatures.

## Material and Methods

### Patient Characteristics and Integrated RNA Profiles

The clinical and raw RNA expression data for 355 patients with GBM (i.e., TCGA GBM cohort) and 10 patients affected by epilepsy (i.e., TCGA normal cohort) were obtained from the TCGA data portal (http://tcga-data.nci.nih.gov/tcga/) in February 2013. Raw data of RNA expression were preprocessed and log2 transformed, and the probe-centric signals were converted to gene-centric signals using the Affy [18] and AgiMicroRNA [19] packages in R software. Detailed methods for sample selection, raw data preprocessing, and profile integration are described in Material S1.

### Statistical Analyses

Univariate survival analysis was performed for preliminary screening of clinical variables that were correlated with the OS of patients with GBM. We also used general multivariate stepwise Cox regression analysis to evaluate the contribution of patient age, KPS, radiotherapy, and targeted molecular therapy as independent clinical prognostic factors. For GBM patients, OS time was calculated from the date of the initial pathologic diagnosis and the date of decease.

The association between continuous RNA expression and OS was preliminarily assessed using univariate Cox regression followed by the Benjamini–Hochberg method [20] for multiple test false discovery rate correction. The RNAs that were differentially expressed between the TCGA GBM cohort and the TCGA normal cohort were further selected by Liu's method [21] with a fold-change >2.0 and adjusted *P* value <0.05. Using these preliminary significant RNAs, we carried out elastic net–regulated Cox regression [15]–[17] with 10,000 iterations and 10 cross–validations to select the variables (i.e., RNAs) and estimate the regression parameters accurately. RNAs with elastic–net regulated Cox regression coefficients ≠ 0 were included in the integrated prognostic RNA signature. The RNAs in the integrated RNA signature with HR>1 were defined as risky RNAs for GBM, and those with HR<1 were defined as protective RNAs.

A prognosis index (PI) for each GBM patient was calculated as a linear combination of the relative expression levels of the RNAs in the integrated RNA signature weighted by their elastic net–regulated Cox regression coefficients. A weighted prognostic index (WPI) defined as the standard form of the PI was adopted for GBM patient stratification. Specifically:
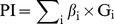


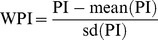
where, 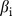 is the elastic net–regulated Cox regression coefficient of the *i*th RNA, 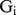 is the relative expression level of the *i*th RNA, mean(PI) and sd(PI) are mean and standard deviation of the PI vector, respectively. GBM patients were assigned into the high- or low-risk groups according to WPI. The Kaplan-Meier method was adopted to test the prognostic value of the integrated RNA signature in GBM, and the log-rank method was used for the survival distribution difference test.

The ability and efficiency of the integrated RNA signature to predict GBM patient outcome was assessed by the area under the curve (AUC) of the receiver-operator characteristic (ROC). Analysis was conducted by the survivalROC package in R software [22]. A permutation test with 10,000 permutations was adopted to test the significance of the observed AUC for the ROC curve (See Material S1 for details on the permutation test).We also compared the prognostic ability of the integrated RNA signature with clinical variables, mRNA-only, and miRNA-only signatures [8], [9]. All analyses were performed using R software (version 2.15.3) [23] and Bioconductor (version 2.11) [24].

### Validation of the Prognostic Value of the Integrated RNA Signature

Five independent external genome-wide expression datasets, with a total of 201 GBM patients, were used to provide robustness and reproducibility for validation of the prognostic value of the integrated RNA signature obtained from the TCGA GBM cohort. One dataset from Freije et al. (n = 58, GSE4412) [25], one from Phillips et al. (n = 54, GSE4271) [26], and three from Lee Y et al. (n = 89, GSE13041) [27], [28] —all of which were generated on the Affymetrix U133A platform—were collected from Gene Expression Omnibus (http://www.ncbi.nlm.nih.gov/geo/) and preprocessed with the Affy package. The clinical data for the validation datasets are listed in Table S3.

### Gene Ontology and Pathway Analysis

We analyzed the gene ontology (GO) with use of the online tool DAVID [29] (http://david.abcc.ncifcrf.gov/). Ingenuity Pathway Analysis (IPA) was employed to obtain information on the relationships, biological mechanisms and pathways of the genes corresponding to the integrated RNA signature for GBM prognosis.

## Results

### Demographic and Clinical Correlation of Patients in the TCGA GBM Cohort

At the time of last follow-up for patients in the TCGA GBM cohort, 289 patients had died due to the disease, 65 were alive, and 1 had been lost to follow-up. The mean age of the patients in the TCGA GBM cohort was 57.47 years, and the median survival time was 428 days (95% confidence interval [CI], 385–463 days). Demographic and clinical data for this cohort are summarized in Table S1. As we expected, age, KPS, radiotherapy, chemotherapy, tumor status, and targeted molecular therapy were significantly correlated with GBM patient survival (Table S1 and Fig. S1). Among these factors, age, KPS, radiotherapy, and targeted molecular therapy were independent factors that correlated with GBM patient survival by multivariate Cox regression (Table S1). The results of this preliminary assessment indicated that the survival data for the TCGA GBM cohort, although containing censored data, were informative and appropriate for use to identify a molecular prognostic signature.

### Integrated Prognostic RNA Model Construction in the TCGA GBM Cohort

Two hundred and twenty RNAs (adjusted *P* value <0.05) were preliminarily identified by univariate Cox regression. Sixty-nine RNAs were selected for further analysis (Table S2) by filtering out the RNAs that were not differentially expressed in GBM. Elastic net–regulated Cox regression resulted in the selection of 16 mRNAs and 1 miRNA (Table 1 and Fig. S2) into the integrated prognostic RNA signature.

**Table 1:** The 17-RNA signature predictive of GBM patient survival.

No.	Gene symbol	HR	Univariate Cox *P* value	BH adjusted *P* value	Permutation test *P* value
**Down-regulated, protective RNAs**					
1	*PCSK1N*	0.868	5.65e-04	0.044	0.000
2	*KATNB1*	0.637	9.31e-05	0.017	0.000
3	*DLGAP1*	0.651	3.16e-04	0.031	0.000
4	*CISD1*	0.623	2.94e-05	0.011	0.000
**Up-regulated, risky RNAs**					
5	*LMAN2*	1.544	8.76e-04	0.049	1.00e-04
6	*IGFBP2*	1.175	8.00e-05	0.015	0.000
7	*CTNNA1*	1.794	8.07e-05	0.015	0.000
8	*P4HB*	1.645	6.54e-05	0.014	0.000
9	*IQCG*	1.239	1.24e-04	0.020	0.000
10	*FAM46A*	1.290	8.18e-05	0.015	1.00e-04
11	*SLC25A20*	1.390	4.77e-05	0.013	0.000
12	*STAT3*	1.559	4.49e-04	0.038	0.000
13	*FMOD*	1.199	3.56e-06	0.006	0.000
14	*ATP13A3*	1.595	2.61e-05	0.011	0.000
15	*EFEMP2*	1.297	1.34e-06	0.004	0.000
16	*BZW1*	1.964	1.98e-04	0.024	0.000
17	*hsa-miR-148a*	1.148	5.74e-05	0.013	0.000

Protective RNAs (HR>1) were down-regulated and risky RNAs (HR<1) were up-regulated in GBM versus normal control. These 17 RNAs correlated with the OS of GBM patients by elastic net–regulated Cox regression. BH, Benjamini-Hochberg method.

**Figure 1:**
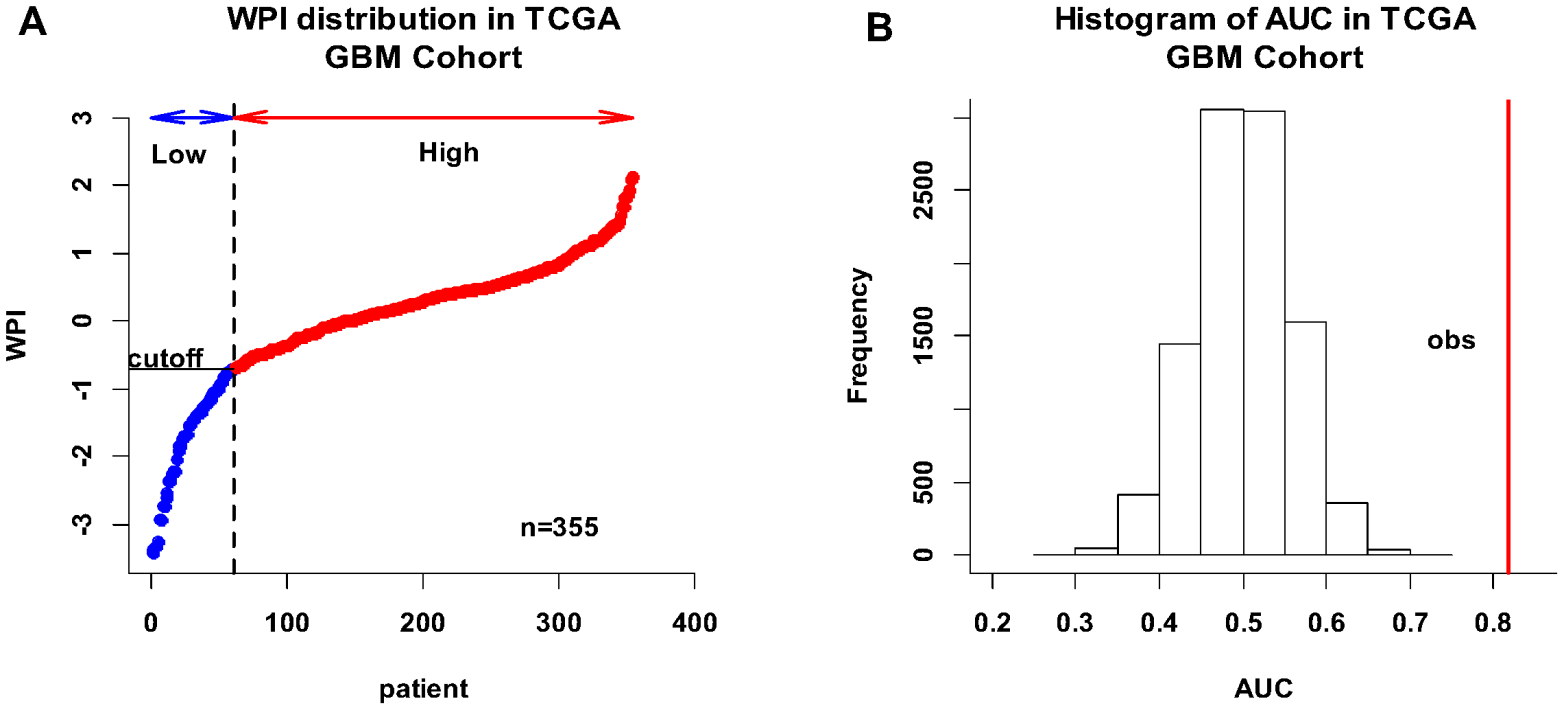
WPI distribution and AUC histogram of the TCGA GBM cohort. A, WPI distribution in the TCGA GBM cohort (n = 355). The point at which the distribution changed the most abruptly, which corresponded to (WPI = −0.7), served as the distribution cutoff. Patients were categorized as high risk (n = 294, left double-headed arrow) or low risk (n = 61, right double-headed arrow). B, Histogram of the empirical distribution of AUC generated from 10,000 permutations. The vertical dashed line is the observed AUC in the TCGA GBM cohort.

**Figure 2:**
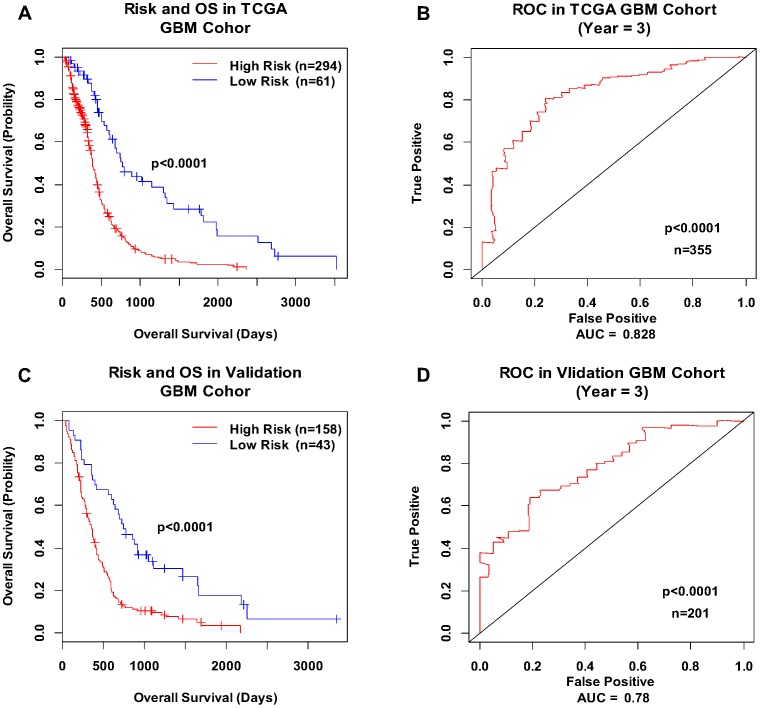
Kaplan-Meier curves and ROC curves for the integrated RNA signature. Kaplan-Meier plots for GBM patients in high-risk and low-risk groups segregated by the integrated RNA signature in the TCGA GBM cohort (A) and the validation cohort (C). The significance of survival difference between groups was evaluated by log-rank test (*P* = 1.02e-09 and 3.76e-08, respectively). The respective ROC curves had AUCs of 0.828 (B) and 0.780 (D). The permutation *P* value was computed to test the null hypothesis (AUC = 0.5) using 10,000 permutations.

As a continuous variable, the PI was significantly correlated with GBM patient survival (HR = 4.596; 95% CI = 3.108–6.796; *P* = 2.13e-14 by Wald test). The WPIs in the TCGA GBM cohort ranged from −3.431 to 2.124 (Fig. 1A). Because GBM is a vicious tumor and has a poor outcome, most patients with GBM are at high risk of death, so to determine how to distribute our cohort into high-risk and low-risk groups, we chose the point at which the distribution changed the most abruptly (i.e., WPI = −0.7) as the cutoff. As a result, the high-risk and low-risk groups consisted of 294 and 61 patients, respectively (Fig. 1A). Survival analysis showed that the median survival time was significantly shorter in the high-risk group than in the low-risk group (339.5 versus 596 days; HR = 2.864; 95% CI = 2.016–4.068; *P* = 4.24e-09 by Wald test; Fig. 2A). In all of the survival analyses, fewer events occurred after 3 years, so we tested the ability of the integrated RNA signature to predict the survival outcome of GBM patients at, and around, this time point. The AUC was 0.828 at 3 years of OS in the TCGA GBM cohort (Fig. 2B). The permutation test resulted in a *P* value of <0.0001 (Fig. 1B). Multivariate stepwise Cox regression analysis revealed that PI, age, KPS, radiotherapy, and targeted molecular therapy were independent prognostic predictors for GBM patient survival. The HR (HR = 4.167) for the integrated RNA signature was greater than that of the demographic and clinical variables, and it implied that the integrated RNA signature had superior performance compared with traditional clinical variables (Table 2).

**Table 2:** Multivariate Cox stepwise regression of PI and demographic and clinical variables.

Variable	HR	95% CI	*P* value
**PI**	4.167	2.551–6.808	1.20e-08
**Age**	1.017	1.005–1.029	0.004
**KPS**	0.989	0.979–1.000	0.047
**Radiotherapy**	0.401	0.238–0.674	0.001
**Targeted molecular therapy**	0.619	0.446–0.859	0.004

The PI based on the integrated 17-RNA signature was an independent prognostic predictor for GBM patients relative to the demographic and clinical variables, and it was superior to clinical variables in predicting GBM patient survival. The significance of the regression model was evaluated by Wald test (*P* = 8.88e-16).

### Cell Death and Survival Network

GO annotation for the mRNA component of the integrated RNA signature revealed that these genes are highly enriched in regulation of growth (GO: 0040008) and response to estradiol stimulus (GO: 0032355). Network analysis revealed that the 16 genes are only involved in cell death and survival, tumor morphology, and cellular development network (score = 49; Fig. S3). The prognostic genes building relationships with each other mainly crossed *TP53*, *STAT3*, *IGFBP2*, and *UBC*. Of these four genes, *TP53*, *STAT3*, and *IGFBP2* are very important regulators for cell growth, cell death, and survival, which suggested that this pathway plays important roles in long-term survival for the low-risk GBM patient group. It is well known that increased tumor cell growth shortens GBM patient survival. Therefore, the candidate genes are suitable for GBM prognosis.

Due to the elastic net variable selection algorithm combines the superiorities of both LASSO regression for dimension reduction and ridge regression for handling colinearity and the colinearity between miRNA and its targets. Thus, targets of *hsa-miR-148a* in the prognostic RNA signature may exist. Using TargetScan software (http://www.targetscan.org/) [30], we found that *DLGPA1* was a potential target of *hsa-miR-148a* (Fig. S3). The expression of *DLGPA1* and *hsa-miR-148a* was negatively correlated in the TCGA GBM cohort (*P* = 0.0002 and 0.0005 by Pearson and Spearman correlation tests, respectively) but was not correlated in the TCGA normal cohort (*P* = 0.821 and 0.967 by Pearson and Spearman correlation tests, respectively), implying that the interaction between *hsa-miR-148a* and *DLGPA1* may exist in GBM patients but not in control subjects. In addition, IPA results revealed that *DLGAP1* is regulated by *TP53* and that *hsa-miR-148a* is indirectly associated with cell growth and cell survival pathways through chemical molecules and protein complex. Thus, *hsa-miR-148a* regulation of *DLGAP1* may play an important role in predicting GBM patient survival.

### Prognostic Value of the Integrated RNA Signature in the Validation GBM Cohort

Because of insufficient published datasets that have both mRNA and miRNA profiles for the same GBM patients and because our integrated prognostic RNA signature consisted almost entirely of mRNAs, we assembled mRNA expression data from five external independent datasets that were generated from the Affymetrix U133A platform to validate the robustness and reproducibility of the prognostic value for the mRNA component of the RNA prognostic signature.

The five expression datasets were combined and called the validation GBM cohort (n = 201 samples). Raw CEL files were preprocessed by the Affy package with the same parameters as described for the TCGA GBM cohort, and the expression matrix of the 16 mRNAs was generated. In the setting of 16 variables (i.e., mRNAs) and 201 samples, the sample size was enough to accurately estimate the regression coefficients by ridge regression. Using the same cutoff value (WPI = −0.7), 158 GBM patients from the validation cohort were classified as high risk and 43 as low risk. Survival analysis showed that GBM patients in the low-risk group lived significantly longer than patients in the high-risk group (HR = 2.453; 95% CI = 1.662–3.634; Wald test *P* = 3.84e-06; Fig. 2C). The AUC of the ROC curve for the mRNA component of the integrated RNA signature was 0.780 at 3 years in the validation GBM cohort (Fig. 2D). The HR calculated based on the integrated RNA signature in the TCGA GBM cohort was 2.864, When the *hsa-miR-148a* was removed from this signature, the value dropped to 2.625. The HR in the validation cohort (HR = 2.453) was less than the HR in the TCGA GBM cohort and close to the HR with *hsa-miR-148a* removed in the TCGA GBM cohort.

### Comparison of Prognostic Ability of the Integrated RNA Signature and Other Prognostic mRNA or miRNA Signatures

We compared the prognostic value of the integrated prognostic RNA signature with two external prognostic signatures, a 10-miRNA model [9] and a 14-mRNA model [8], that have been used for risk stratification of GBM patients.

GBM patient risk stratification based on the 10-miRNA model followed by survival analysis showed a significant survival difference between the high-risk and low-risk patient groups in the TCGA GBM cohort (HR = 2.135; 95% CI = 1.601–2.856; Wald test *P* = 2.79e-07; Fig. S4A). Similarly, for the 14-mRNA model, the OS was significantly poorer for GBM patients in the high-risk group than for patients in the low-risk group in the TCGA GBM cohort (HR = 1.946; 95% CI = 1.443–2.681; *P* = 2.17e-05 by Wald test; Fig. S4B). The AUC of the ROC curve for each model in the TCGA GBM cohort at 3 years was 0.757 and 0.742, respectively (Fig. S4C and Fig. S4D). These results validated the prognostic value of the 10-miRNA model [9] and the 14-mRNA mode [8]. Multivariate stepwise Cox regression that included the PI we generated from the integrated RNA signature and the PIs from the two external RNA models as covariates showed that all three models were found to be independent significant predictors for GBM patient survival. The HRs corresponding to the integrated RNA, 10-miRNA, and 14-mRNA models were 2.689, 1.673 and 1.520, respectively (Table 3), which implied that our integrated RNA signature was superior to the mRNA-only model or the miRNA-only model for predicting GBM patient survival. GBM patient risk stratification based on the 14-mRNA model followed by survival analysis showed a significant survival difference between the high-risk and low-risk patient groups in the validation GBM cohort (HR = 1.919; 95% CI = 1.358–2.711; Wald test *P* = 2.2e-04). Multivariate Cox regression with PIs generated from the 14-mRNA model and 16-mRNA model in the validation cohort shows that both models are independent significant factors for GBM prognosis (Table S4). However, the hazard ratio (HR) of the 16-mRNA model is greater than the 14-mRNA model which implies the 16-mRNA model also superior to the 14-mRNA model in the validation cohort.

**Table 3:** Multivariate Cox stepwise regression of PIs of three RNA prognostic models of GBM patient survival.

PI	HR	95% CI	*P* value
17-RNA model	2.689	1.651–4.382	7.13e-05
10-miRNA model	1.673	1.180–2.373	0.004
14-mRNA model	1.520	1.041–.217	0.030

All three models were significant predictors of GBM patient survival, but the integrated 17-RNA model was superior. The significance of the regression model was evaluated by Wald test (*P* = 6.22e-15).

## Discussion

In this study, we proposed a novel integrated mRNA and miRNA signature including that predicting GBM patient survival more accurately than clinical parameters, mRNA-only signature, and miRNA-only signature. Moreover, we were able to validate the prognostic value of our signature in five additional datasets of GBM. Pathway analysis revealed that genes in our signature were involved in cell death and survival. All results suggested that the integrated signature was suitable and had superior prognostic value for GBM patient prognosis. In addition, a potential interaction between *hsa-miR-148a* and DLGAP1 correlated with GBM patient survival was identified. Our study provides novel insights into the significance of molecular markers in predicting the prognosis of GBM patients.

Although markers for classifying GBM molecular subtypes have been identified [10], markers associated mainly with the pathogenesis of GBM may be not prognostic. For example, in the analysis of Verhaak et al. of the TCGA data, biologically based subtypes were not prognostic of GBM patient outcome [10]. In contrast, we identified common RNAs that consistently drive the outcome for GBM patients irrespective of the clinical or molecular subtype. For GBM prognostic markers discovery, studies have been focused on either mRNA [7], [8] or miRNA [9] profiles and an integrated RNA analysis is in need.

Of the prognostic genes, *IGFBP2*, *EFEMP2*, *SLC25A20*, *FMOD*, *BZW1*, and *STAT3* were previously reported to be associated with GBM patient survival [31]–[36]. To the best of our knowledge, we are the first to report the other 11 candidate genes. Consistent with the GO result, we found that *IGFBP2*, *STAT3* and *CISD1* were involved in the regulation of the growth process, which plays a vital role in GBM malignancy. *CISD1* plays a key role in regulating maximal capacity for electron transport and oxidative phosphorylation; decreased *CISD1* induces the dysregulation of electron transport and oxidative phosphorylation [37]. In our study, the expression of *CISD1* was down-regulated, by which we infer that lower expression of *CISD1* might be due to glycolysis enhancement that could suppress oxidative phosphorylation (i.e., the Warburg effect).

Among the risky genes, *CTNNA1*, *P4HB*, and *LMAN2* are associated with tumor development. *CTNNA1* plays a crucial role in cell differentiation and is over-expressed in GBM [38]. Recent research has found that *P4HB* is over-expressed in temozolomide-resistant GBM cells [39], so its up-regulation may confer drug resistance in GBM. *LMAN2* is known to be over-expressed in gastric cancer [40], and we found it was over-expressed in GBM as well. *LMAN2* mainly participates in the early secretory pathway and is involved in glycosylation alteration by sorting glycoproteins carrying high mannose-type glycans, which is a general feature of cancer cells [41]. Although the sugar-binding properties of this gene has been characterized in detail, its biological function in GBM has not yet been identified. The roles of the rest of the up-regulated genes identified–*IQCG*, *FAM46A*, and *ATP13A3*–in GBM remain unclear.

We identified three protective genes that were down-regulated in GBM patients. *KATNB1* mainly participates in the disassembly of microtubules, and it may be involved in tumor development [42]. We found *KATNB1* down-regulated in our analysis and down-regulation of *KATNB1* would decrease its ability to disassemble microtubules, thereby keeping tumor cells alive. *PCSK1N* functions in the control of the neuroendocrine secretory pathway and is a potent inhibitor of PC1/3 [43], [44], but its role in GBM is unclear. *DLGAP1* is part of the postsynaptic scaffold in neuronal cells. Down-regulation of *DLGAP1* has reported in colorectal tumor [45]. The decreased *DLGAP1* expression in tumor is mainly due to hypermethylation and that *DLGAP1* might play a growth-suppressive role in colorectal tumor [45].

Finally, we found that *hsa-miR-148a* was inversely correlated with GBM patients' survival. This miRNA was previously reported by other researchers to be up-regulated in GBM [46], [47], although how it might be associated with tumorigenesis and patient survival, its function, and its biological process in GBM are unclear. Only one suggestion has been made, that *hsa-miR-148a* is oncogenic in GBM [48]. Interestingly, we found that *DLGAP1* was not only one of the target genes of *hsa-miR-148a* but also a prognostic gene in our study. The dysregulation of both *hsa-miR-148a* and *DLGAP1* may be an important predictor of GBM patient survival.

In conclusion, we have identified a 17-RNA integrated signature that can predict the survival outcome of GBM patients more accurately than previously developed mRNA or miRNA signatures have. Our findings may help researchers understanding of GBM cell death and survival, develop targeted therapy, and identify high-risk GBM patients for better disease management.

## Supporting Information

Figure S1 **Kaplan-Meier OS curves for demographic and clinical variables in the TCGA GBM cohort.** Age (in years) at initial pathologic diagnosis (A), KPS (B), patient tumor status (C), chemotherapy (D), radiotherapy (E), and targeted molecular therapy (F) were each statistically significant variables (*P*<0.05 by log-rank test) by univariate survival analysis.
(TIF)

Figure S2 **Cross-validation error curve.** The left vertical line shows where the cross-validation error curve hits its minimum (lambda  = 0.56). The right vertical line shows the most regularized model with cross-validation error within 1 standard deviation of the minimum. The minimum was achieved by a fairly regularized model (n = 17), but the right line indicates that the null model (no coefficients included) is within 1 standard deviation of the minimum. The numbers at the top of the figure indicate the number of nonzero coefficients.
(TIF)

Figure S3 **Cell death and survival, tumor morphology, and cellular development network.** Schematic representation of the most significant network for the integrated RNA signature using IPA. This network had a high score of 49. Green and red nodes represent down-regulated and up-regulation genes, respectively. The red line between *has-miR-148a* and *DLGAP1* represented negative regulation that predicted by TargetScan.
(TIF)

Figure S4 **Kaplan-Meier OS curves and ROC curves for the 10-miRNA and 14 mRNA prognostic signatures.** High-risk and low-risk patients in the TCGA GBM cohort were segregated by the 10-miRNA (A) or 14-mRNA (B) signature. The significance of the survival difference between groups was evaluated using the log-rank test (*P* = 8.35e-07 and 6.13e-05, respectively). The ROC curves had AUCs of 0.757 (C) and 0.742 (D).
(TIF)

Table S1 **Survival analysis of patients in the TCGA GBM cohort (n = 355), by demographic and clinical variables.** Variables with *P*<0.05 by log-rank test were considered statistically significant and variables with *P*<0.05 by multivariate Cox regression were considered as independent clinical variables for GBM patient prognosis. The significance of the multivariate Cox regression model was evaluated by Wald test (*P* = 2.41e-10).
(DOCX)

Table S2 **RNAs preliminarily selected by univariate Cox regression (n = 69).** RNAs up-regulated and risky (HR>1) or down-regulated and protective (HR<1) in GBM versus normal control. NA, not available.
(DOCX)

Table S3 **Demographic characteristics of patients of the validation GBM cohort (n = 201).** Vital status denotes patient survival outcome at the last follow up: 1, deceased and 0, alive.
(DOCX)

Table S4 **Multivariate Cox stepwise regression of PIs generated from the 16-mRNA model and the 14-mRNA model in the validation GBM cohort.**
(DOCX)

Material S1 **Supporting material.**
(DOC)
