# Supplementary figures and images for: An Integrated mRNA and microRNA Expression Signature for Glioblastoma Multiforme Prognosis
Source: PLoS One. 2014 May 28;9(5):e98419. doi: 10.1371/journal.pone.0098419 (PMC4037214; doi:10.1371/journal.pone.0098419)

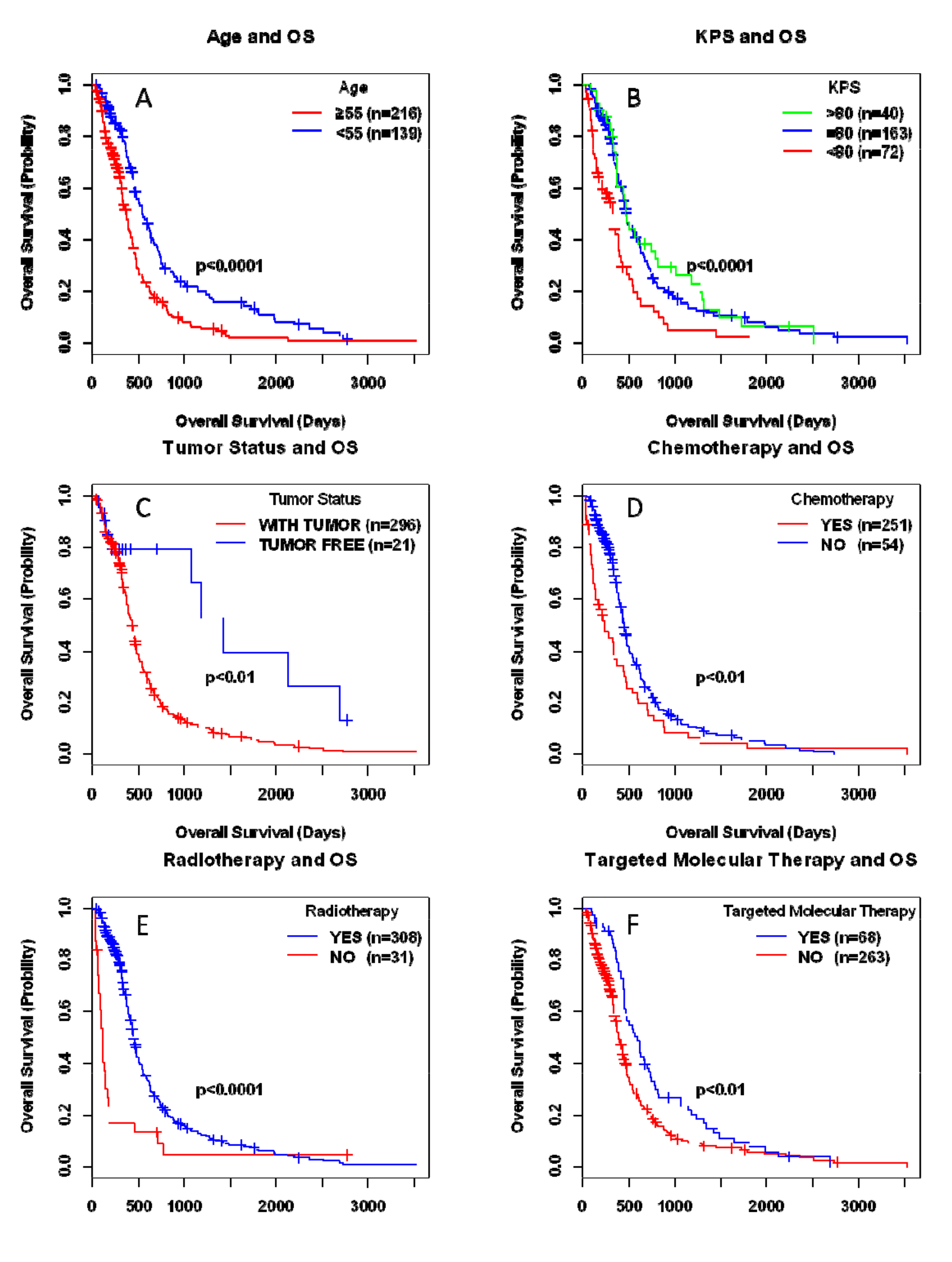

Supplement: Figure S1 — Kaplan-Meier OS curves for demographic and clinical variables in the TCGA GBM cohort. Age (in years) at initial pathologic diagnosis (A), KPS (B), patient tumor status (C), chemotherapy (D), radiotherapy (E), and targeted molecular therapy (F) were each statistically significant variables (P<0.05 by log-rank test) by univariate survival analysis. (TIF) [file pone.0098419.s001.tif]

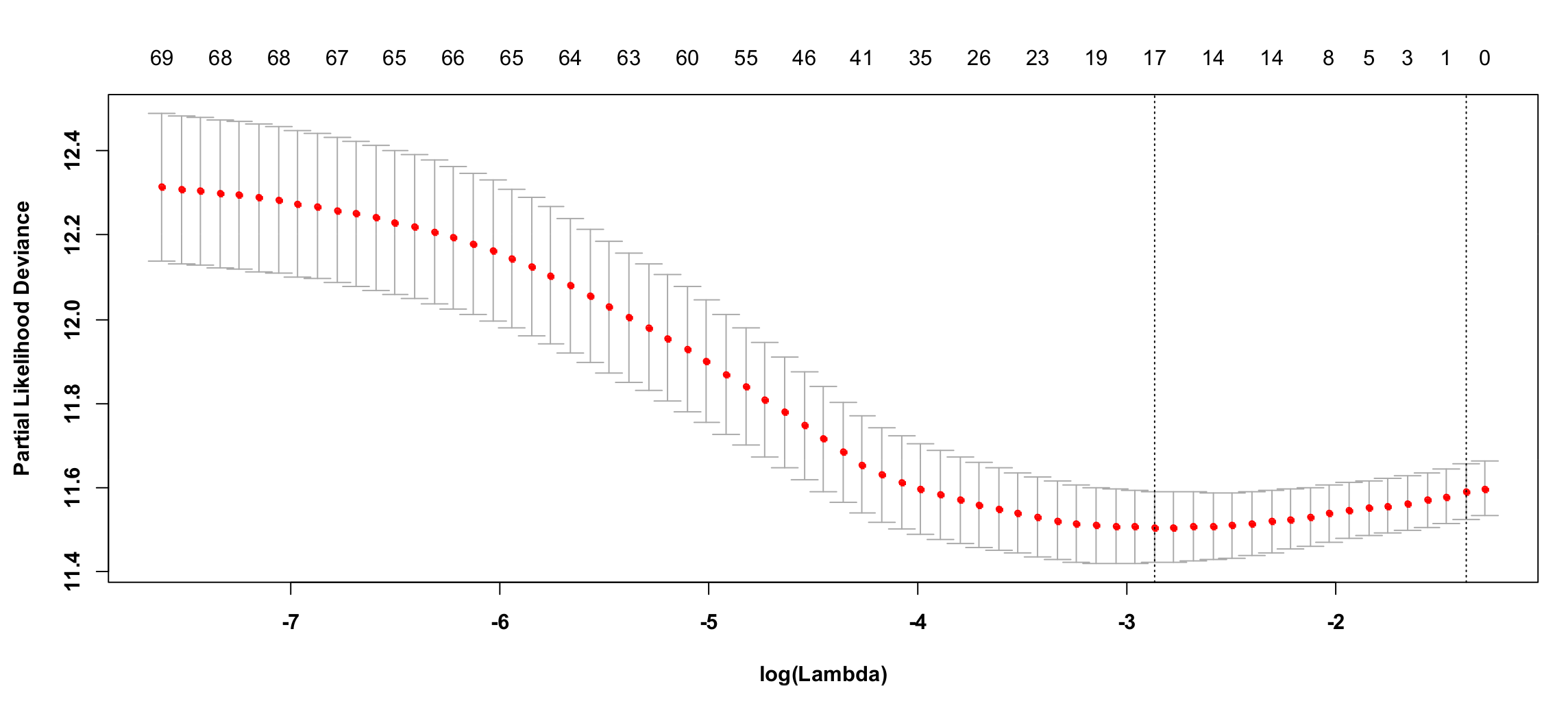

Supplement: Figure S2 — Cross-validation error curve. The left vertical line shows where the cross-validation error curve hits its minimum (lambda = 0.56). The right vertical line shows the most regularized model with cross-validation error within 1 standard deviation of the minimum. The minimum was achieved by a fairly regularized model (n = 17), but the right line indicates that the null model (no coefficients included) is within 1 standard deviation of the minimum. The numbers at the top of the figure indicate the number of nonzero coefficients. (TIF) [file pone.0098419.s002.tif]

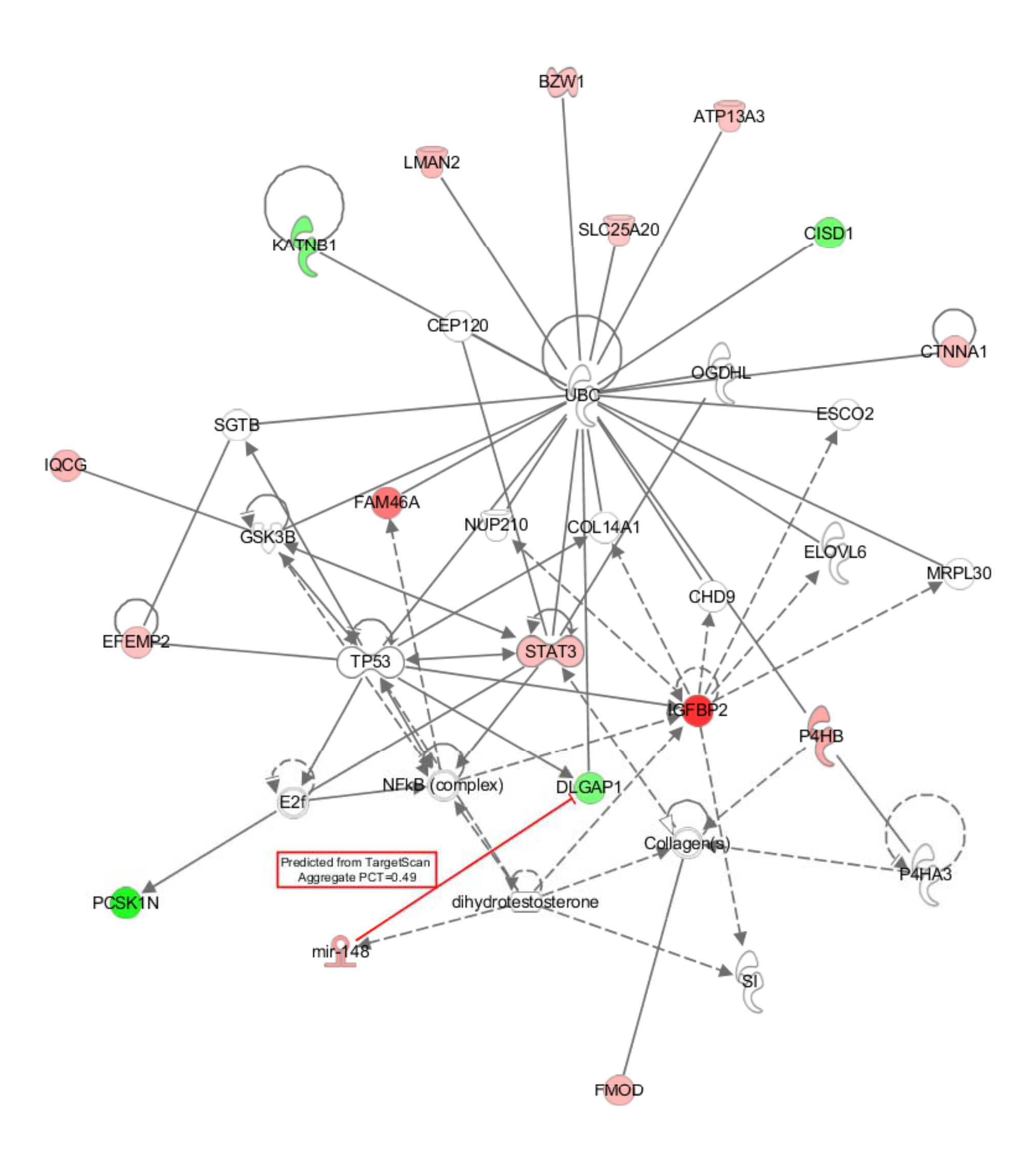

Supplement: Figure S3 — Cell death and survival, tumor morphology, and cellular development network. Schematic representation of the most significant network for the integrated RNA signature using IPA. This network had a high score of 49. Green and red nodes represent down-regulated and up-regulation genes, respectively. The red line between has-miR-148a and DLGAP1 represented negative regulation that predicted by TargetScan. (TIF) [file pone.0098419.s003.tif]

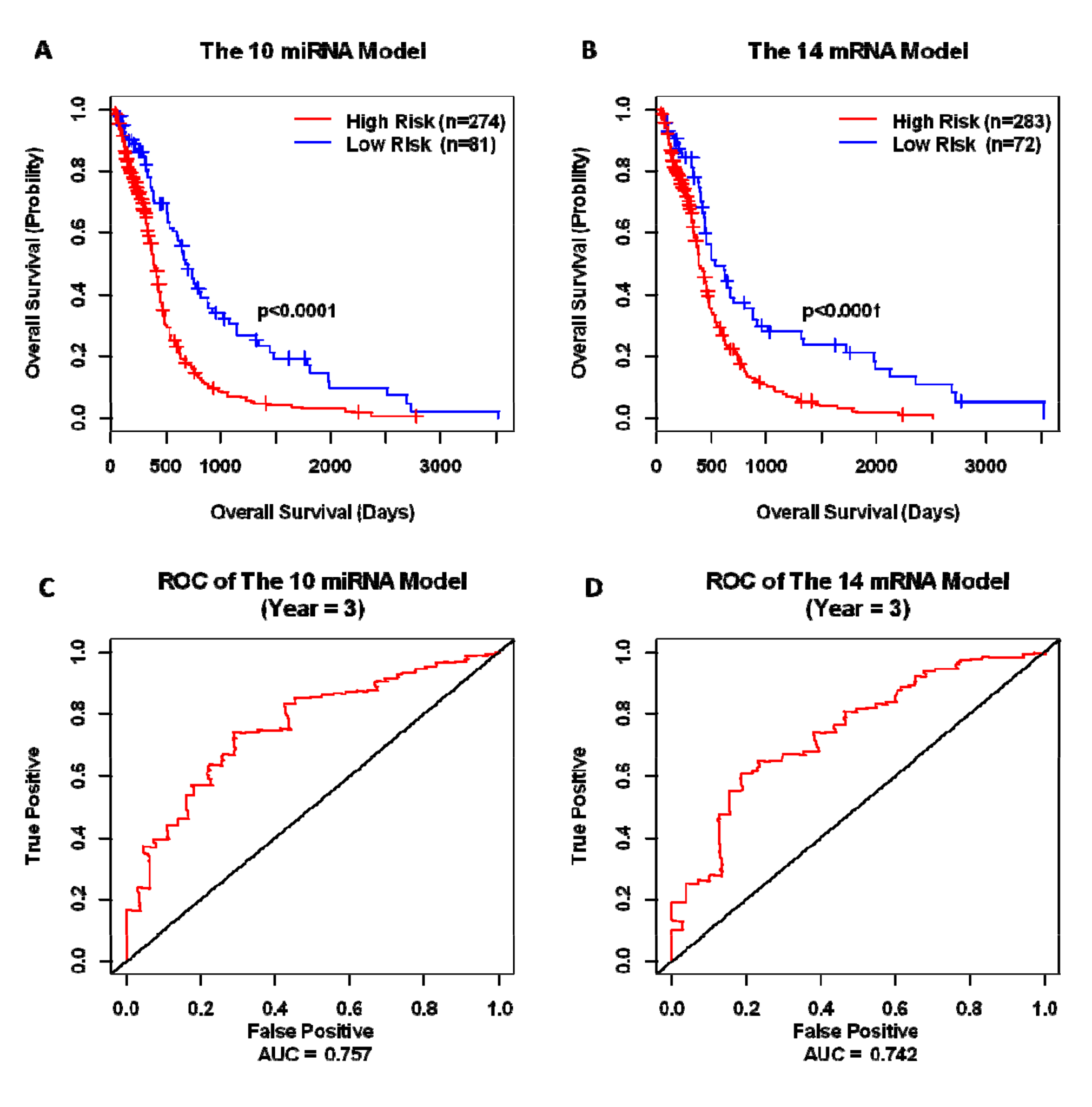

Supplement: Figure S4 — Kaplan-Meier OS curves and ROC curves for the 10-miRNA and 14 mRNA prognostic signatures. High-risk and low-risk patients in the TCGA GBM cohort were segregated by the 10-miRNA (A) or 14-mRNA (B) signature. The significance of the survival difference between groups was evaluated using the log-rank test (P = 8.35e-07 and 6.13e-05, respectively). The ROC curves had AUCs of 0.757 (C) and 0.742 (D). (TIF) [file pone.0098419.s004.tif]
