# Supplementary material for: An Integrated mRNA and microRNA Expression Signature for Glioblastoma Multiforme Prognosis
Source: PLoS One. 2014 May 28;9(5):e98419. doi: 10.1371/journal.pone.0098419 (PMC4037214; doi:10.1371/journal.pone.0098419)
Supplement: Table S1 — Survival analysis of patients in the TCGA GBM cohort (n = 355), by demographic and clinical variables. Variables with P<0.05 by log-rank test were considered statistically significant and variables with P<0.05 by multivariate Cox regression were considered as independent clinical variables for GBM patient prognosis. The significance of the multivariate Cox regression model was evaluated by Wald test (P = 2.41e-10). (DOCX) [file pone.0098419.s005.docx]

**Table S1. Survival analysis of patients in the TCGA GBM cohort (n = 355), by demographic and clinical variables**

| **Variable** | **Count** | **Percentage** | **Median survival time** | **95% CI** | **Log-rank test *P* value** | **Multivariate Cox regression *P* value** |
| --- | --- | --- | --- | --- | --- | --- |
| **Age** |  |  |  |  |  |  |
| ≥55 years | 216 | 60.85% | 372 | (327,415) | 5.67e-07 | 3.74e-05 |
| <55 years | 139 | 39.15% | 540 | (484,648) |  |  |
| **KPS** |  |  |  |  |  |  |
| >80 | 40 | 11.27% | 459 | (372,811) | 2.17e-05 | 0.027 |
| =80 | 164 | 46.20% | 479 | (432,559) |  |  |
| <80 | 72 | 20.28% | 337 | (213,394) |  |  |
| **Tumor Status** |  |  |  |  |  |  |
| WITH TUMOR | 296 | 83.38% | 432 | (385,466) | 2.02e-03 | 0.830 |
| TUMOR FREE | 21 | 5.92% | 1426 | (1068,1123) |  |  |
| **Chemotherapy** |  |  |  |  |  |  |
| YES | 251 | 70.70% | 451 | (418,485) | 3.81e-03 | 0.336 |
| NO | 54 | 15.21% | 231 | (139,438) |  |  |
| **Radiotherapy** |  |  |  |  |  |  |
| YES | 308 | 86.76% | 451 | (422,485) | 2.74e-07 | 1.41e-06 |
| NO | 31 | 8.73% | 111 | (83,164) |  |  |
| **Targeted molecular therapy** |  |  |  |  |  |  |
| YES | 68 | 19.15% | 587 | (463,728) | 3.51e-03 | 0.006 |
| NO | 263 | 74.08% | 386 | (360,438) |  |  |
| **Immunotherapy** |  |  |  |  |  |  |
| YES | 10 | 2.82% | 547 | (463,456) | 0.610 |  |
| NO | 310 | 87.32% | 422 | (378,453) |  |  |
| **Hormonal therapy** |  |  |  |  |  |  |
| YES | 51 | 14.37% | 485 | (418,587) | 0.606 |  |
| NO | 271 | 76.34% | 415 | (372,452) |  |  |
| **Prior glioma** |  |  |  |  |  |  |
| YES | 9 | 2.54% | 542 | (372,438) | 0.397 |  |
| NO | 346 | 97.46% | 427 | (385,459) |  |  |
| **Gender** |  |  |  |  |  |  |
| MALE | 221 | 62.25% | 427 | (384,467) | 0.986 |  |
| FEMALE | 134 | 37.75% | 447 | (360,532) |  |  |

Variables with *P* < 0.05 by log-rank test were considered statistically significant and variables with *P* < 0.05 by multivariate Cox regression were considered as independent clinical variables for GBM patient prognosis. The significance of the multivariate Cox regression model was evaluated by Wald test (*P* = 2.41e-10).
