# Supplementary material for: An Integrated mRNA and microRNA Expression Signature for Glioblastoma Multiforme Prognosis
Source: PLoS One. 2014 May 28;9(5):e98419. doi: 10.1371/journal.pone.0098419 (PMC4037214; doi:10.1371/journal.pone.0098419)
Supplement: Table S2 — RNAs preliminarily selected by univariate Cox regression (n = 69). RNAs up-regulated and risky (HR>1) or down-regulated and protective (HR<1) in GBM versus normal control. NA, not available. (DOCX) [file pone.0098419.s006.docx]

**Table S2. RNAs preliminarily selected by univariate Cox regression (n = 69)**

| **No.** | **Gene symbol** | **HR** | **Univariate Cox *P* value** | **BH-adjusted *P* value** | **Fold change (logged)** | **Description** |
| --- | --- | --- | --- | --- | --- | --- |
| 1 | *BASP1* | 0.86 | 7.11e-04 | 0.045 | -3.10 | brain abundant, membrane attached signal protein 1 |
| 2 | *PCSK1N* | 0.87 | 5.65e-04 | 0.044 | -2.11 | proprotein convertase subtilisin/kexin type 1 inhibitor |
| 3 | *ZNF804A* | 0.83 | 5.97e-04 | 0.044 | -1.84 | zinc finger protein 804A |
| 4 | *ZNF248* | 0.73 | 1..78e-04 | 0.023 | -1.70 | zinc finger protein 248 |
| 5 | *REPS2* | 0.71 | 5.71e-04 | 0.044 | -1.63 | RALBP1 associated Eps domain containing 2 |
| 6 | *R3HDM1* | 0.61 | 4.28e-04 | 0.038 | -1.61 | R3H domain containing 1 |
| 7 | *IL1RAPL1* | 0.64 | 4.00e-04 | 0.036 | -1.57 | interleukin 1 receptor accessory protein-like 1 |
| 8 | *CISD1* | 0.62 | 2.94e-05 | 0.011 | -1.31 | CDGSH iron sulfur domain 1 |
| 9 | *DLGAP1* | 0.65 | 3.16e-04 | 0.031 | -1.24 | discs, large (Drosophila) homolog-associated protein 1 |
| 10 | *KATNB1* | 0.64 | 9.31e-05 | 0.017 | -1.24 | katanin p80 (WD repeat containing) subunit B 1 |
| 11 | *NMT2* | 0.73 | 7.19e-04 | 0.045 | -1.09 | N-myristoyltransferase 2 |
| 12 | *PPA1* | 0.64 | 4.82e-05 | 0.013 | -1.08 | pyrophosphatase (inorganic) 1 |
| 13 | *SLC25A20* | 1.39 | 4.77e-05 | 0.013 | 1.01 | solute carrier family 25 (carnitine/acylcarnitine translocase), member 20 |
| 14 | *SDF4* | 1.79 | 1.05e-05 | 0.018 | 1.01 | stromal cell derived factor 4 |
| 15 | *PLA2G5* | 1.12 | 2.16e-05 | 0.025 | 1.05 | phospholipase A2, group V |
| 16 | *BZW1* | 1.96 | 1.98e-04 | 0.024 | 1.11 | basic leucine zipper and W2 domains 1 |
| 17 | *ATP13A3* | 1.59 | 2.61e-05 | 0.011 | 1.13 | ATPase type 13A3 |
| 18 | *FKBP9* | 1.39 | 8.29e-06 | 0.009 | 1.14 | FK506 binding protein 9, 63 kDa |
| 19 | *DNAJC10* | 1.36 | 7.51e-04 | 0.046 | 1.14 | DnaJ (Hsp40) homolog, subfamily C, member 10 |
| 20 | *CTNNA1* | 1.79 | 8.07e-05 | 0.015 | 1.16 | catenin (cadherin-associated protein), alpha 1, 102kDa |
| 21 | *EFEMP2* | 1.30 | 1.34e-06 | 0.004 | 1.16 | EGF containing fibulin-like extracellular matrix protein 2 |
| 22 | *MR1* | 1.36 | 6.37e-04 | 0.044 | 1.16 | major histocompatibility complex, class I-related |
| 23 | *HSPA5* | 1.56 | 3.72e-05 | 0.011 | 1.17 | heat shock 70kDa protein 5 (glucose-regulated protein, 78kDa) |
| 24 | *BST2* | 1.18 | 3.78e-04 | 0.035 | 1.18 | bone marrow stromal cell antigen 2 |
| 25 | *STAT3* | 1.56 | 4.49e-04 | 0.038 | 1.18 | signal transducer and activator of transcription 3 (acute-phase response factor) |
| 26 | *KIAA0226L* | 1.21 | 1.59e-04 | 0.022 | 1.21 | KIAA0226-like |
| 27 | *LMAN2* | 1.54 | 8.76e-04 | 0.049 | 1.28 | lectin, mannose-binding 2 |
| 28 | *IQCG* | 1.24 | 1.24e-04 | 0.020 | 1.33 | IQ motif containing G |
| 29 | *FMOD* | 1.20 | 3.56e-06 | 0.006 | 1.33 | fibromodulin |
| 30 | *LGALS3* | 1.17 | 3.81e-04 | 0.035 | 1.36 | lectin, galactoside-binding, soluble, 3 |
| 31 | *RNASE4* | 1.25 | 2.46e-04 | 0.027 | 1.39 | ribonuclease, RNase A family, 4 |
| 32 | *SLC27A3* | 1.24 | 2.26e-04 | 0.026 | 1.41 | solute carrier family 27 (fatty acid transporter), member 3 |
| 33 | *GANAB* | 1.38 | 7.38e-04 | 0.046 | 1.41 | glucosidase, alpha; neutral AB |
| 34 | *CLEC2B* | 1.16 | 6.78e-04 | 0.045 | 1.44 | C-type lectin domain family 2, member B |
| 35 | *P4HB* | 1.64 | 6.54e-05 | 0.014 | 1.57 | prolyl 4-hydroxylase, beta polypeptide |
| 36 | *ANG* | 1.23 | 7.37e-04 | 0.046 | 1.58 | angiogenin, ribonuclease, RNase A family, 5 |
| 37 | *CLEC5A* | 1.18 | 1.43e-04 | 0.022 | 1.65 | C-type lectin domain family 5, member A |
| 38 | *UPP1* | 1.19 | 3.49e-04 | 0.033 | 1.66 | uridine phosphorylase 1 |
| 39 | *SERPING1* | 1.16 | 6.63e-04 | 0.045 | 1.70 | serpin peptidase inhibitor, clade G (C1 inhibitor), member 1 |
| 40 | *DIRAS3* | 1.18 | 3.12e-05 | 0.011 | 1.76 | DIRAS family, GTP-binding RAS-like 3 |
| 41 | *MYD88* | 1.48 | 6.47e-04 | 0.044 | 1.77 | myeloid differentiation primary response gene (88) |
| 42 | *C1RL* | 1.22 | 7.63e-05 | 0.015 | 1.79 | complement component 1, r subcomponent-like |
| 43 | *GNG12* | 1.36 | 5.94e-04 | 0.044 | 1.82 | guanine nucleotide binding protein (G protein), gamma 12 |
| 44 | *DRAM1* | 1.24 | 3.16e-04 | 0.031 | 1.84 | DNA-damage regulated autophagy modulator 1 |
| 45 | *DUSP6* | 1.26 | 1.63e-04 | 0.022 | 1.88 | dual specificity phosphatase 6 |
| 46 | *C1S* | 1.15 | 7.52e-04 | 0.046 | 1.91 | complement component 1, s subcomponent |
| 47 | *FNDC3B* | 1.37 | 3.22e-05 | 0.011 | 1.98 | fibronectin type III domain containing 3B |
| 48 | *AEBP1* | 1.18 | 8.18e-05 | 0.015 | 2.08 | AE binding protein 1 |
| 49 | *SLC43A3* | 1.44 | 9.05e-06 | 0.009 | 2.33 | solute carrier family 43, member 3 |
| 50 | *IL1RAP* | 1.19 | 1.45e-04 | 0.022 | 2.35 | interleukin 1 receptor accessory protein |
| 51 | *SLC2A10* | 1.20 | 1.87e-04 | 0.023 | 2.37 | solute carrier family 2 (facilitated glucose transporter), member 10 |
| 52 | *MSN* | 1.42 | 4.51e-06 | 0.007 | 2.38 | moesin |
| 53 | *NMI* | 1.29 | 4.43e-04 | 0.038 | 2.39 | N-myc (and STAT) interactor |
| 54 | *FAM46A* | 1.29 | 8.18e-05 | 0.015 | 2.50 | family with sequence similarity 46, member A |
| 55 | *TIMP1* | 1.25 | 1.68e-05 | 0.009 | 2.56 | TIMP metallopeptidase inhibitor 1 |
| 56 | *TAGLN2* | 1.29 | 5.91e-05 | 0.013 | 2.58 | transgelin 2 |
| 57 | *ANXA2P2* | 1.23 | 6.03e-04 | 0.044 | 2.61 | annexin A2 pseudogene 2 |
| 58 | *EMP3* | 1.21 | 1.33e-05 | 0.009 | 2.63 | epithelial membrane protein 3 |
| 59 | *C1R* | 1.18 | 1.85e-04 | 0.023 | 2.63 | complement component 1, r subcomponent |
| 60 | *ANXA2* | 1.23 | 5.24e-04 | 0.042 | 2.72 | annexin A2 |
| 61 | *PYGL* | 1.23 | 3.33e-04 | 0.032 | 2.78 | phosphorylase, glycogen, liver |
| 62 | *WWTR1* | 1.25 | 4.44e-04 | 0.038 | 2.99 | WW domain containing transcription regulator 1 |
| 63 | *CHI3L1* | 1.10 | 7.74e-04 | 0.047 | 3.21 | chitinase 3-like 1 (cartilage glycoprotein-39) |
| 64 | *PDPN* | 1.16 | 4.44e-05 | 0.012 | 3.45 | podoplanin |
| 65 | *IGFBP2* | 1.17 | 8.00e-05 | 0.015 | 3.77 | insulin-like growth factor binding protein 2, 36kDa |
| 66 | *PTX3* | 1.11 | 8.89e-04 | 0.049 | 3.80 | pentraxin 3, long |
| 67 | *CD44* | 1.24 | 7.18e-04 | 0.045 | 4.66 | CD44 molecule (Indian blood group) |
| 68 | *hsa-miR-34a* | 1.18 | 3.04e-04 | 0.031 | 1.68 | NA |
| 69 | *hsa-miR-148a* | 1.15 | 5.74e-05 | 0.013 | 1.85 | NA |

RNAs up-regulated and risky (HR > 1) or down-regulated and protective (HR < 1) in GBM versus normal control. NA, not available.
