# Supplementary material for: An Integrated mRNA and microRNA Expression Signature for Glioblastoma Multiforme Prognosis
Source: PLoS One. 2014 May 28;9(5):e98419. doi: 10.1371/journal.pone.0098419 (PMC4037214; doi:10.1371/journal.pone.0098419)
Supplement: Table S3 — Demographic characteristics of patients of the validation GBM cohort (n = 201). Vital status denotes patient survival outcome at the last follow up: 1, deceased and 0, alive. (DOCX) [file pone.0098419.s007.docx]

**Table S3. Demographic characteristics of patients of the validation GBM cohort (n = 201)**

| **No.** | **Name** | **Survival time (days)** | **Vital status** | **Age** | **Sex** | **GEO accession no.** |
| --- | --- | --- | --- | --- | --- | --- |
| 1 | GBM 712 | 188 | 1 | 49 | M | GSM99432 |
| 2 | GBM 932 | 98 | 1 | 18 | F | GSM99434 |
| 3 | GBM 697 | 356 | 1 | 64 | F | GSM99436 |
| 4 | GBM 931 | 182 | 1 | 58 | F | GSM99438 |
| 5 | GBM 1656 | 961 | 0 | 48 | M | GSM99440 |
| 6 | GBM 1902 | 325 | 1 | 56 | F | GSM99442 |
| 7 | GBM 2015 | 396 | 0 | 78 | M | GSM99444 |
| 8 | GBM 1667 | 588 | 1 | 44 | M | GSM99446 |
| 9 | GBM 1745 | 780 | 0 | 42 | F | GSM99448 |
| 10 | GBM 1511 | 85 | 1 | 27 | M | GSM99450 |
| 11 | GBM 706 | 506 | 1 | 54 | M | GSM99452 |
| 12 | GBM 995 | 56 | 1 | 76 | F | GSM99454 |
| 13 | GBM 1495 | 71 | 1 | 82 | F | GSM99456 |
| 14 | GBM 749 | 53 | 1 | 39 | M | GSM99462 |
| 15 | GBM 1028 | 54 | 1 | 20 | F | GSM99464 |
| 16 | GBM 1414 | 64 | 1 | 69 | F | GSM99466 |
| 17 | GBM 1342 | 224 | 1 | 54 | M | GSM99470 |
| 18 | GBM 2158 | 95 | 1 | 62 | F | GSM99472 |
| 19 | GBM 1463 | 265 | 1 | 30 | M | GSM99474 |
| 20 | GBM 636 | 412 | 1 | 54 | F | GSM99476 |
| 21 | GBM 824 | 186 | 1 | 50 | F | GSM99478 |
| 22 | GBM 839 | 286 | 1 | 82 | M | GSM99480 |
| 23 | GBM 1032 | 90 | 1 | 34 | F | GSM99482 |
| 24 | GBM 1900 | 279 | 1 | 45 | F | GSM99484 |
| 25 | GBM 2079 | 298 | 0 | 64 | M | GSM99486 |
| 26 | GBM 1544 | 148 | 1 | 49 | F | GSM99488 |
| 27 | GBM 1398 | 293 | 1 | 49 | F | GSM99490 |
| 28 | GBM 1423 | 96 | 1 | 54 | F | GSM99492 |
| 29 | GBM 1469 | 153 | 1 | 56 | M | GSM99494 |
| 30 | GBM 746 | 43 | 1 | 52 | M | GSM99524 |
| 31 | GBM 996 | 224 | 1 | 45 | M | GSM99526 |
| 32 | GBM 1334 | 302 | 1 | 36 | F | GSM99528 |
| 33 | GBM 2028 | 223 | 1 | 42 | F | GSM99530 |
| 34 | GBM 2029 | 223 | 1 | 42 | F | GSM99532 |
| 35 | GBM 938 | 569 | 1 | 29 | M | GSM99536 |
| 36 | GBM 2067 | 168 | 1 | 42 | F | GSM99538 |
| 37 | GBM 2068 | 168 | 1 | 42 | F | GSM99540 |
| 38 | GBM 1406 | 236 | 1 | 29 | M | GSM99542 |
| 39 | GBM 1022 | 126 | 1 | 31 | M | GSM99544 |
| 40 | GBM 660 | 112 | 1 | 75 | F | GSM99546 |
| 41 | GBM 976 | 418 | 1 | 43 | F | GSM99548 |
| 42 | GBM 1354 | 420 | 1 | 42 | M | GSM99550 |
| 43 | GBM 1478 | 1088 | 0 | 40 | M | GSM99552 |
| 44 | GBM 1516 | 1031 | 0 | 41 | F | GSM99554 |
| 45 | GBM 1798 | 723 | 0 | 47 | F | GSM99556 |
| 46 | GBM 2166 | 1098 | 0 | 39 | F | GSM99558 |
| 47 | GBM 1043 | 1089 | 1 | 23 | F | GSM99560 |
| 48 | GBM 1521 | 1022 | 0 | 23 | F | GSM99562 |
| 49 | GBM 1675 | 683 | 1 | 61 | M | GSM99564 |
| 50 | GBM 604 | 185 | 1 | 66 | M | GSM99572 |
| 51 | GBM 782 | 140 | 1 | 24 | F | GSM99576 |
| 52 | GBM 2017 | 237 | 1 | 60 | M | GSM99578 |
| 53 | GBM 1038 | 1247 | 0 | 33 | F | GSM99580 |
| 54 | GBM 1905 | 389 | 1 | 75 | M | GSM99582 |
| 55 | GBM 2098 | 203 | 0 | 65 | M | GSM99584 |
| 56 | GBM 585 | 302 | 1 | 63 | F | GSM99586 |
| 57 | GBM 1681 | 927 | 0 | 40 | M | GSM99588 |
| 58 | GBM 2013 | 396 | 0 | 70 | F | GSM99590 |
| 59 | PRB3910 | 84 | 1 | 43 | F | GSM96996 |
| 60 | PRB3836 | 112 | 1 | 34 | F | GSM96984 |
| 61 | PRB3827 | 224 | 1 | 51 | F | GSM96979 |
| 62 | PRB3834 | 224 | 1 | 54 | M | GSM96983 |
| 63 | PRB3776 | 224 | 1 | 59 | M | GSM96967 |
| 64 | PRB3775 | 231 | 1 | 44 | M | GSM96966 |
| 65 | PRB3895 | 231 | 1 | 34 | M | GSM96987 |
| 66 | PRB3749 | 238 | 1 | 49 | M | GSM96955 |
| 67 | PRB3906 | 273 | 1 | 32 | F | GSM96994 |
| 68 | PRB4125 | 287 | 1 | 30 | F | GSM97007 |
| 69 | PRB4094 | 357 | 1 | 32 | F | GSM96997 |
| 70 | PRB3782 | 364 | 1 | 76 | M | GSM96973 |
| 71 | PRB4126 | 371 | 1 | 39 | M | GSM97008 |
| 72 | PRB4785 | 371 | 1 | 54 | M | GSM97040 |
| 73 | PRB4787 | 371 | 1 | 54 | M | GSM97041 |
| 74 | PRB3778 | 385 | 1 | 82 | M | GSM96969 |
| 75 | PRB3833 | 392 | 1 | 48 | F | GSM96982 |
| 76 | PRB4127 | 399 | 1 | 72 | M | GSM97009 |
| 77 | PRB3897 | 399 | 1 | 55 | M | GSM96988 |
| 78 | PRB3779 | 413 | 1 | 41 | M | GSM96970 |
| 79 | PRB3759 | 413 | 1 | 49 | M | GSM96964 |
| 80 | PRB3757 | 434 | 1 | 54 | M | GSM96962 |
| 81 | PRB3900 | 434 | 1 | 53 | F | GSM96990 |
| 82 | PRB4097 | 434 | 1 | 57 | M | GSM97000 |
| 83 | PRB4122 | 455 | 1 | 72 | F | GSM97004 |
| 84 | PRB3748 | 490 | 1 | 45 | M | GSM96954 |
| 85 | PRB4128 | 490 | 1 | 57 | M | GSM97010 |
| 86 | PRB4782 | 511 | 1 | 49 | F | GSM97037 |
| 87 | PRB3832 | 539 | 1 | 47 | M | GSM96981 |
| 88 | PRB3907 | 553 | 1 | 58 | F | GSM96995 |
| 89 | PRB4102 | 637 | 1 | 68 | F | GSM97002 |
| 90 | PRB3825 | 665 | 1 | 40 | M | GSM96977 |
| 91 | PRB3786 | 679 | 1 | 54 | M | GSM96976 |
| 92 | PRB3774 | 679 | 1 | 48 | M | GSM96965 |
| 93 | PRB3899 | 742 | 1 | 49 | M | GSM96989 |
| 94 | PRB3839 | 777 | 1 | 48 | M | GSM96985 |
| 95 | PRB3784 | 861 | 1 | 29 | M | GSM96974 |
| 96 | PRB3905 | 875 | 1 | 54 | M | GSM96993 |
| 97 | PRB3830 | 917 | 1 | 68 | F | GSM96980 |
| 98 | PRB3744 | 917 | 1 | 60 | M | GSM96950 |
| 99 | PRB4753 | 1015 | 0 | 24 | M | GSM97018 |
| 100 | PRB3901 | 1050 | 1 | 36 | F | GSM96991 |
| 101 | PRB3903 | 1078 | 0 | 48 | M | GSM96992 |
| 102 | PRB3753 | 1267 | 1 | 57 | M | GSM96958 |
| 103 | PRB3758 | 1421 | 1 | 34 | M | GSM96963 |
| 104 | PRB3781 | 1470 | 0 | 43 | F | GSM96972 |
| 105 | PRB3747 | 1470 | 1 | 38 | M | GSM96953 |
| 106 | PRB3745 | 1652 | 1 | 48 | F | GSM96951 |
| 107 | PRB3754 | 1666 | 1 | 55 | M | GSM96959 |
| 108 | PRB3826 | 1694 | 0 | 57 | M | GSM96978 |
| 109 | PRB4747 | 1939 | 0 | 22 | F | GSM97014 |
| 110 | PRB3756 | 2177 | 1 | 50 | M | GSM96961 |
| 111 | PRB3746 | 2191 | 1 | 43 | M | GSM96952 |
| 112 | PRB4788 | 2254 | 1 | 45 | M | GSM97042 |
| 113 | TB_115 | 147 | 1 | 67 | F | GSM326790 |
| 114 | TB_686 | 364 | 1 | 50 | M | GSM326791 |
| 115 | TB_1015 | 271 | 1 | 68 | M | GSM326792 |
| 116 | TB_1522 | 591 | 1 | 54 | M | GSM326793 |
| 117 | TB_383 | 207 | 1 | 67 | M | GSM326794 |
| 118 | TB_564 | 241 | 1 | 57 | M | GSM326795 |
| 119 | TB_906 | 109 | 1 | 57 | M | GSM326796 |
| 120 | TB_959 | 184 | 1 | 60 | F | GSM326797 |
| 121 | TB_1129 | 290 | 1 | 59 | M | GSM326798 |
| 122 | TB_1143 | 606 | 1 | 61 | F | GSM326799 |
| 123 | TB_1166 | 600 | 1 | 66 | F | GSM326800 |
| 124 | TB_1170 | 40 | 1 | 58 | M | GSM326801 |
| 125 | TB_1271 | 41 | 1 | 66 | M | GSM326802 |
| 126 | TB_1288 | 233 | 1 | 58 | F | GSM326803 |
| 127 | TB_156 | 591 | 1 | 57 | M | GSM326804 |
| 128 | TB_192 | 263 | 1 | 76 | M | GSM326805 |
| 129 | TB_69 | 646 | 1 | 58 | F | GSM326806 |
| 130 | TB_711 | 1696 | 1 | 71 | M | GSM326807 |
| 131 | TB_883 | 583 | 1 | 70 | M | GSM326808 |
| 132 | TB_917 | 488 | 1 | 55 | M | GSM326809 |
| 133 | TB_943 | 731 | 1 | 56 | M | GSM326810 |
| 134 | TB_987 | 290 | 1 | 63 | M | GSM326811 |
| 135 | TB_999 | 47 | 1 | 76 | M | GSM326812 |
| 136 | TB_1005 | 858 | 1 | 57 | F | GSM326813 |
| 137 | TB_1079 | 218 | 1 | 79 | F | GSM326814 |
| 138 | TB_1091 | 710 | 1 | 53 | M | GSM326815 |
| 139 | TB_1406 | 115 | 1 | 75 | F | GSM326816 |
| 140 | TB_70 | 923 | 1 | 53 | F | GSM326817 |
| 141 | TB_956 | 710 | 1 | 58 | M | GSM326818 |
| 142 | TB_966 | 579 | 1 | 66 | M | GSM326819 |
| 143 | TB_1297 | 559 | 1 | 72 | F | GSM326820 |
| 144 | GBM 1039 | 1110 | 1 | 30 | M | GSM326707 |
| 145 | GBM 1489 | 338 | 1 | 55 | M | GSM326708 |
| 146 | GBM 1796 | 203 | 1 | 42 | M | GSM326709 |
| 147 | GBM 1825 | 447 | 1 | 61 | M | GSM326710 |
| 148 | GBM 2371 | 1249 | 0 | 44 | M | GSM326711 |
| 149 | GBM 658 | 3353 | 0 | 48 | M | GSM326712 |
| 150 | GBM 681 | 49 | 1 | 55 | M | GSM326713 |
| 151 | GBM 751 | 1215 | 1 | 55 | F | GSM326714 |
| 152 | GBM 1388 | 414 | 1 | 34 | M | GSM326715 |
| 153 | GBM 1735 | 73 | 1 | 53 | F | GSM326716 |
| 154 | GBM 2434 | 263 | 1 | 77 | M | GSM326717 |
| 155 | GBM 1823 | 596 | 1 | 47 | M | GSM326718 |
| 156 | GBM 1481 | 2222 | 0 | 78 | F | GSM326719 |
| 157 | GBM 1507 | 358 | 1 | 46 | M | GSM326720 |
| 158 | GBM 1659 | 123 | 1 | 58 | M | GSM326721 |
| 159 | GBM 1671 | 622 | 1 | 70 | F | GSM326722 |
| 160 | GBM 1696 | 172 | 1 | 40 | M | GSM326723 |
| 161 | GBM 1725 | 211 | 1 | 60 | F | GSM326724 |
| 162 | GBM 1782 | 181 | 1 | 40 | M | GSM326725 |
| 163 | GBM 1817 | 763 | 1 | 56 | F | GSM326726 |
| 164 | GBM 2036 | 1473 | 0 | 46 | F | GSM326727 |
| 165 | GBM 2117 | 219 | 1 | 46 | M | GSM326728 |
| 166 | GBM 2149 | 334 | 1 | 46 | M | GSM326729 |
| 167 | GBM 2456 | 376 | 1 | 59 | M | GSM326730 |
| 168 | GBM 2819 | 406 | 1 | 45 | M | GSM326731 |
| 169 | GBM 608 | 380 | 1 | 69 | F | GSM326732 |
| 170 | GBM 628 | 153 | 1 | 76 | M | GSM326733 |
| 171 | GBM 673 | 553 | 1 | 41 | M | GSM326734 |
| 172 | GBM 00AX | 619 | 1 | 39 | M | GSM326821 |
| 173 | GBM 00BP | 222 | 1 | 81 | F | GSM326822 |
| 174 | GBM 00DJR | 599 | 1 | 43 | F | GSM326823 |
| 175 | GBM 00ICR | 728 | 1 | 39 | M | GSM326824 |
| 176 | GBM 00PA | 362 | 1 | 60 | F | GSM326825 |
| 177 | GBM 00QP | 621 | 1 | 63 | F | GSM326826 |
| 178 | GBM 00JS | 79 | 1 | 69 | F | GSM326827 |
| 179 | GBM 00KN | 308 | 1 | 54 | F | GSM326828 |
| 180 | GBM 00LK | 295 | 1 | 64 | F | GSM326829 |
| 181 | GBM 00ME | 1644 | 1 | 49 | M | GSM326830 |
| 182 | GBM 00MO | 506 | 1 | 33 | F | GSM326831 |
| 183 | GBM 00XP | 192 | 1 | 49 | F | GSM326832 |
| 184 | GBM 00KY | 586 | 1 | 40 | F | GSM326834 |
| 185 | GBM 00MF | 494 | 1 | 47 | M | GSM326835 |
| 186 | GBM 00BR | 209 | 1 | 85 | F | GSM326836 |
| 187 | GBM 0092987 | 691 | 1 | 45 | F | GSM326837 |
| 188 | GBM 00DT | 561 | 1 | 56 | M | GSM326838 |
| 189 | GBM 00EM | 359 | 1 | 56 | M | GSM326839 |
| 190 | GBM 00EX | 313 | 1 | 58 | M | GSM326840 |
| 191 | GBM 00FM | 622 | 1 | 55 | M | GSM326841 |
| 192 | GBM 00IA | 322 | 1 | 68 | M | GSM326842 |
| 193 | GBM 00IM | 118 | 1 | 59 | F | GSM326843 |
| 194 | GBM 00KE | 408 | 1 | 43 | M | GSM326844 |
| 195 | GBM 00LO | 37 | 1 | 86 | M | GSM326845 |
| 196 | GBM 00MN | 181 | 1 | 46 | M | GSM326846 |
| 197 | GBM 00MW | 95 | 1 | 71 | F | GSM326847 |
| 198 | GBM 00TIR | 365 | 1 | 54 | M | GSM326848 |
| 199 | GBM 00EJ | 360 | 1 | 56 | M | GSM326849 |
| 200 | GBM 00IN | 141 | 1 | 70 | M | GSM326850 |
| 201 | GBM 00IW | 144 | 1 | 59 | M | GSM326851 |

Vital status denotes patient survival outcome at the last follow up: 1, deceased and 0, alive.
