# Supplementary material for: An Integrated mRNA and microRNA Expression Signature for Glioblastoma Multiforme Prognosis
Source: PLoS One. 2014 May 28;9(5):e98419. doi: 10.1371/journal.pone.0098419 (PMC4037214; doi:10.1371/journal.pone.0098419)
Supplement: Table S4 — Multivariate Cox stepwise regression of PIs generated from the 16-mRNA model and the 14-mRNA model in the validation GBM cohort. (DOCX) [file pone.0098419.s008.docx]

**Table S4**. **Multivariate Cox stepwise regression of PIs generated from the 16-mRNA model and the 14-mRNA model in the validation GBM cohort**

| **Variable** | **HR** | **95% CI** | ***P* value** |
| --- | --- | --- | --- |
| PI (16 mRNAs) | 3.798 | 2.354- 6.129 | 4.61e-08 |
| PI(14 mRNAS) | 2.4733 | 1.129- 5.417 | 0.0236 |
