# Supplementary material for: An Integrated mRNA and microRNA Expression Signature for Glioblastoma Multiforme Prognosis
Source: PLoS One. 2014 May 28;9(5):e98419. doi: 10.1371/journal.pone.0098419 (PMC4037214; doi:10.1371/journal.pone.0098419)
Supplement: Material S1 — Supporting material. (DOC) [file pone.0098419.s009.doc]

**Supplementary Material**

**Sample Selection**

Tumor samples from 355 patients with GBM (i.e., TCGA GBM cohort) were selected by the following criteria: (1) the patients had not received pretreatment, (2) the patients had fully characterized mRNA (HT-HG-U133A) and miRNA (miRNA_8x15k) profiles of tumors, (3) the patients were still alive at least 1 month after initial pathologic diagnosis, (4) the average percentage of necrosis in samples was <40% on both the top and bottom slides, and (5) the average of normal cells and stromal cells in samples was <10% on both the top and bottom slides.

**Preprocessing and Integration of miRNA and mRNA Profiles of the TCGA GBM Cohort**

The mRNA raw CEL files were subjected to background correction in the RMA algorithm, quantiles normalized and medianpolish summarized to generate the log-transformed relative expression for probesets using the Affy package in R software. The probesets that presented in at least one sample were selected by the function mas5calls. To reduce multiple probesets per gene, for each gene we chose the probeset with the highest intensity as the best representation. After filtering, 12,061 probesets (mRNA) were included in the integrated RNA profiles. Raw data of miRNA were analyzed with the AgiMicroRNA package in R software using the RMA algorithm implemented in the Affy package to generate the normalized signal. Control features and miRNAs that were not detected in any sample were filtered with the function filterMicroRna of AgiMicroRNA package. The known batch effects were removed by “Combat” function incorporated in “sva” R package. Finally, 12,310 RNAs that included 12,061 mRNAs and 249 miRNAs were integrated to identify RNA signature for GBM patient prognosis.

**Permutation Test**

The permutation test is a widely applicable non-parametric test. It is most useful when the researcher has insufficient information about the distribution of the data, is uncomfortable making assumptions about the distribution, or cannot easily compute the distribution of the test statistic. The permutation test uses random shuffles of the data to obtain the correct distribution of a test statistic under a null hypothesis. In this study, we sampled the survival time and survival status of GBM patients 10,000 times to generate the empirical distributions of the univariate Cox regression coefficient and the AUC of the ROC curve. The permutation *P* value was calculated as the count of shuffled univariate Cox regression coefficients or shuffled AUCs of the ROCs that exceeded the observed coefficients or AUCs divided by 10,000.
